# Antimicrobial Properties of Spent Hops Extracts, Flavonoids Isolated Therefrom, and Their Derivatives

**DOI:** 10.3390/molecules23082059

**Published:** 2018-08-17

**Authors:** Agnieszka Bartmańska, Ewa Wałecka-Zacharska, Tomasz Tronina, Jarosław Popłoński, Sandra Sordon, Ewa Brzezowska, Jacek Bania, Ewa Huszcza

**Affiliations:** 1 Department of Chemistry, Wrocław University of Environmental and Life Sciences, Norwida 25, 50-375 Wrocław, Poland; tomasz.tronina@upwr.edu.pl (T.T.); jaroslaw.poplonski@upwr.edu.pl (J.P.); sandra.sordon@upwr.edu.pl (S.S.); ewa.brzezowska@upwr.edu.pl (E.B.); ewa.huszcza@upwr.edu.pl (E.H.); 2 Department of Food Hygiene and Consumer Health Protection, Wrocław University of Environmental and Life Sciences, Norwida 25, 50-375 Wrocław, Poland; ewa.walecka@upwr.edu.pl (E.W.-Z.); jacek.bania@upwr.edu.pl (J.B.)

**Keywords:** spent hops, extracts, flavonoids, antibacterial activity, antifungal activity

## Abstract

Hop cones preparations possess a wide range of biological activities including antimicrobial properties. In this work, we evaluated the effect of various organic extracts obtained from spent hops, as well as six hops flavonoids and their twenty natural and synthetic derivatives on human and plant microbial pathogens. Methylene chloride, acetone, ethyl acetate, and methanol were used as extractants. Seven flavonoids, among them two natural (α,β-dihydroxanthohumol and 8-prenylnaringenin) showed significant activity against methicillin sensitive and resistant *Staphylococcus aureus* and *Staphylococcus epidermidis* strains with the lowest MIC80 value of 0.5 µg/mL. The crude ethyl acetate, acetone, and methanol extracts from the spent hops exhibited antifungal activity against *Fusarium oxysporum*, *F. culmorum,* and *F. semitectum* with the lowest MIC50 of 0.5 mg/mL, while the methylene chloride extract exerted antifungal activity against *Botrytis cinerea* with the MIC50 of 1 mg/mL. The preparation obtained after the removal of xanthohumol from the spent hops crude extracts retained up to 95% of activity. These findings suggest that various spent hops extracts may be effective agents for the control of plant pathogens of economic importance, like *Botrytis cinerea* and *Fusarium oxysporum*, while some compounds from spent hops or their derivatives may become useful for staphylococcal infections.

## 1. Introduction

Numerous studies have demonstrated at the laboratory and field level that plant extracts contain diverse bioactive components that can control phytopathogens growth [1]. On the other hand, medicinal plants are a rich source of therapeutic compounds that are of use in the modern pharmaceutical industry, among them as antimicrobials against different types of microbes, including food-borne pathogens [2,3,4]. For ecological and economic reasons, the interest in plant waste as a source of compounds with antimicrobial activity has significantly increased recently [5,6]. Particular attention is paid to polyphenolic compounds, which, as natural factors that protect plants against infections, often become the subject of anti-infective research [7,8].

The female inflorescens of hop plant (*Humulus lupulus* L., Cannabinaceae) are used primarily in the brewing industry to add bitterness, aroma and flavor to beer. At the beginning of human beer manufacturing, hop cones were added to provide antimicrobial properties in order to preserve the brew. These characteristics are mainly attributed to the presence of bitter acids [9,10]. Before use in the brewing industry, hops were traditionally applied for medicinal purposes, mainly for the treatment of sleeping disorders, activation of gastric functions and as stomachic, antibacterial and antifungal remedies [11]. This information indicates that the hop plant is an excellent source of compounds with antimicrobial activity.

Since large commercial brewers often apply for beer manufacturing hop extracts obtained by the extraction of cones by supercritical carbon dioxide (CO_2_ extracts), it is reasonable to study the antimicrobial formulations derived from spent hops. This waste material contains a large number of valuable flavonoids that are not extracted with carbon dioxide under the conditions used for the production of common hop extracts for the brewing industry. Xanthohumol (**1**) is the principal flavonoid found in the hop plant which is the object of greatest interest to researchers, due to the wide spectrum of biological activity, especially broad-spectrum cancer chemopreventive ones with inhibitory mechanisms in the initiation, promotion and progression phase of carcinogenesis [12,13]. The content of xanthohumol ranges from 0.1 to 1% of the cone dry mass [14]. Except for xanthohumol, in different hop varieties, there are at least 30 other flavonoids present amounting from 10 to 100-fold lower [15]. One of them is 8-prenylnaringenin, the most potent known plant-derived estrogen [16].

Various hops extracts, as well as individual hops compounds, are known for their antimicrobial activity. The hop bitter acids (humulones and lupulones) have shown activity against Gram-positive bacteria i.e. *Lactobacillus*, *Streptococcus*, *Staphylococcus*, *Listeria*, *Clostridium* and *Bacillus* species [17,18,19,20,21], gram-negative bacteria i.e. *Helicobacter pylori* and *Brucella* species [22,23] and some fungi i.e. *Candida*, *Trichophyton*, *Fusarium* and *Mucor* species [10]. This action has been attributed to the interference of a prenyl group of hop acids with the bacterial cell plasma membrane [24].

The same structural element is responsible for the antimicrobial activity of flavonoids found in hops. Prenylflavonoids exhibit antibacterial activities, especially against certain Gram-positive bacteria. Xanthohumol and 6-prenylnaringenin inhibited the growth of *Staphylococcus aureus* [25]. Xanthohumol also demonstrated antibacterial activity against certain oral cavity pathogens from the Streptococcus genus and pathogens caused skin inflammation and acne vulgaris from the genera of *Propionibacterium*, *Staphylococcus*, *Streptococcus*, and *Kocuria* [18,26]. Antifungal activity of prenylflavonoids include the ability of xanthohumol, 6- and 8-prenylnaringenin to inhibit the growth of *Mucor rouxianus* and the dermatophytic fungi *Trichophyton spp* [25].

Although the antimicrobial activity of spent hops extracts was previously examined, none of these studies investigated the impacts of different extraction solvents on the antimicrobial potential of these preparations [27,28]. Therefore, the purpose of this work was to evaluate the antifungal properties of four organic extracts obtained from spent hops, as well as residues of these extracts formed after the removal of xanthohumol. The antibacterial activity of extracts, hops flavonoids and its derivatives were also investigated. To our best knowledge, this is the first study on the antibacterial activity of minor hops flavonoids other than isoxanthohumol, 6- and 8-prenylnaringenin.

## 2. Results

### 2.1. Production of Type I and Type II Extracts

The extraction of the spent hops process with four organic solvents with different polarity yielded in methylene chloride (26.01 g/kg), ethyl acetate (38.57 g/kg), acetone (29.82 g/kg) and methanol (92.95 g/kg) crude extracts, described in this paper as “extracts type I”. The removal of xanthohumol (**1**) from them using Sephadex LH-20 gel chromatography resulted in the so-called “extracts type II”. All the extracts obtained were examined for the content of xanthohumol (**1**), and the results of these analyses are shown in Table 1.

### 2.2. Antibacterial Activity

**Figure 1:**
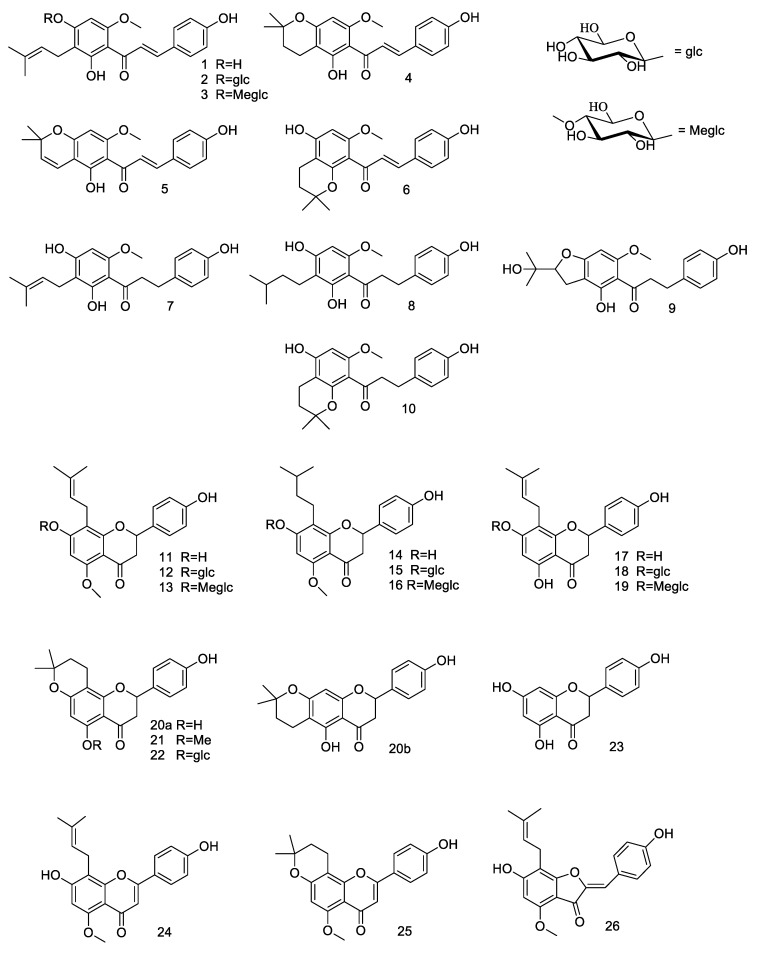
Hops flavonoids (**1**, **4**, **5**, **7, 11**, **17**), their derivatives obtained by microbial transformation (**2**, **3**, **9**, **12**, **13**, **15**, **16**, **18**, **19**, **22**) and by chemical modifications (**6**, **8**, **10**, **14**, **20a**, **20b**, **21**, **24**, **25**, **26**), and model compound naringenin (**23**).

The extracts type I and II as well as the six hops flavonoids (**1**, **4**, **5**, **7**, **11**, **17**), their ten natural derivatives obtained by microbial transformation (**2**, **3**, **9**, **12**, **13**, **15**, **16**, **18**, **19**, **22**), ten synthetic derivatives (**6**, **8**, **10**, **14**, **20a**, **20b**, **21**, **24**, **25**, **26**), and also naringenin (**23**) as a model compound, shown in Figure 1 were tested in vitro for their antibacterial activity. 

In the first approach, the agar diffusion method was used to evaluate the antibacterial activity of the crude extracts and flavonoids at the dose of 100 µg and 50 µg per well against *Staphylococcus aureus* ATCC19095, *Salmonella typhimurium* PCM2665 and *Listeria monocytogenes* ATCC7644. This preliminary assay showed that all four extracts type I and compounds **1**–**8**, **11**, **14**, **17**, mixture of **20a**, **20b**, and **26** exhibited antimicrobial activity against *Staphylococcus aureus* ATCC19095. For substances **7**, **8**, **14**, **17**, mixture of **20a**, **20b** and **26**, the MIC80 values were equal to or less than 50 µg/mL. These results are reported in Table 2.

### 2.3. Antifungal Activity

The antifungal activity of the spent hops extracts, both type I and type II, was evaluated at the concentration of 1 mg/mL against 7 plant pathogenic fungi: *Fusarium culmorum* AM10, *Fusarium equiseti* AM15, *Fusarium semitectum* AM20, *Fusarium oxysporum* AM21, *Botrytis cinerea* AM235, *Penicillium purpurogenum* AM80 and *Mucor hiemalis* AM450. The results of these experiments are presented in Table 3. 

For the four most sensitive fungi, the MIC50 values were determined (Table 4). Amphotericin B and xanthohumol (**1**) were used as positive control.

## 3. Discussion

Limited publications are available on the antimicrobial activity of spent hops extracts and only one of them concerned the effect of solvents used for extraction on antimicrobial activity [19,23,24]. Therefore, we decided to check the usefulness of crude extracts from this cheap waste material derived from the edible plant, which is also commonly used in medical applications.

Following the results of the extracts production, evidently targets methanol as the solvent of choice for the production of a large volume of crude extracts. The increase of polarity of the solvent increases the yield of the extract obtained from spent hops (methylene chloride (26.01 g/kg), ethyl acetate (38.57 g/kg), acetone (29.82 g/kg) and methanol (92.95 g/kg)), which is probably associated with the lower selectivity of extraction or the higher content of polar fraction in the spent hops. This is apparently the result of the supercritical CO_2_ extraction that selectively extracts resinous compounds for beer production, which should be more efficiently extracted with methylene chloride, than with methanol. Since the most abundant of the compounds present in the extracts was xanthohumol (**1**) we decided to evaluate its content in the extracts obtained, with the combination of the further removal of (extracts type II) to exclude its background activity in the extracts. The highest content of xanthohumol (**1**) in the spent hops preparations was observed in the crude acetone extract, less of that flavonoid contained a crude extract of ethyl acetate. However, given the efficiency and selectivity of the extraction of **1**, thus a yield of the total xanthohumol (**1**) extracted with different solvents from spent hops, the best extractant is ethyl acetate (3.51 g/kg), followed by acetone (2.97 g/kg), methanol (2.94 g/kg) and methylene chloride (1.33 g/kg). 

### 3.1. Antibacterial Activity

The antibacterial activity of the extracts and flavonoids was measured by MIC80 determination which is defined as the concentration that inhibits the growth of 80% of organisms. Because the strain *Staphylococcus aureus* ATCC19095 exhibited the highest sensitivity in the agar diffusion test, four additional strains were included to the microbroth dilution assay, namely: methicillin sensitive and resistant strains of the *Staphylococcus* genus: *S. aureus* ATCC29213 (MSSA), *S. aureus* ATCC43300 (MRSA), *S. epidermidis* 91M (MRSE) and *S. epidermidis* 4s (MSSE, enterotoxigenic).

All the selected compounds (**7**, **8**, **14**, **17**, **20a, 20b**, **26**) showed antibacterial activity against the *Staphylococcus* species, none of them inhibited *Listeria monocytogenes* ATCC7644 and only aurone (**26**) was active against *Salmonella typhimurium* PCM2565 with the MIC80 value of 50 µg/mL (Table 2). For all the tested microorganisms, 5% dimethylsulfoxide (negative control) did not affect growth. The lowest MIC80 was observed for α,β,2′,3′-tetrahydroxanthohumol (**8**) and *S. aureus* ATCC19095 (MSSA), being two times lower than the antibiotic used as the positive control (Table 2). Of special interest is the most active natural flavonoid α,β-dihydroxanthohumol (**7**), which inhibited all the tested strains of *Staphylococcus aureus* and *Staphylococcus epidermidis*, both susceptible and resistant to methicillin. The same results were obtained for aurone (**26**), the synthetic derivative of xanthohumol (**1**) (Table 2). 

By comparison of the structure of tested compounds, the antistaphylococcal activity can be related to features such as the leak of a double bond in prenyl group (chalcone **8** and flavanone **14**), hydrogenated α,β double bond in chalcones (**7** and **8**), free C-5 hydoxyl group in flavanones (**17**, **20a**, **20b**) and the aurone skeleton (**26**). According to previous studies, the tested glycosides were not active [29].

The presence of the hydrophobic prenyl group in flavonoids usually increases the antimicrobial activity [30]. This relationship can be observed for 8-prenylnaringenin (**17**) which efficiently inhibited staphylococci, whereas naringenin (**23**) without the prenyl group was inactive. However, in our study, the mixture of compounds **20a, 20b** with the cyclized prenyl group was also characterized by high activity against *S. aureus* and *S. epidermidis*, including methicillin resistant strains. No activity of flavanone **21,** with the C-5 methoxy group in comparison to **20a**, suggests that the free C-5 hydroxyl group in combination with the pyrane ring affords antimicrobial activity. Moreover, the synergism between **20a** and **20b** cannot be excluded. It has been reported that methoxy groups decrease the antibacterial activity of flavonoids against methicillin-resistant *Staphylococcus aureus* strains [31]. It is also in agreement with previous studies on hop flavonoids reporting that the C-5 methoxy group in isoxanthohumol (**11**) decreases their activity against *S. aureus* in comparison to 8-prenylnaringenin (**17**) [25]. In contrast to the results described in that publication, it should be noted that we found 8-prenylnaringenin (**17**) more active against the five *Staphylococcus* strains tested than xanthohumol (**1**), where the MIC80 value was in all cases greater than 50 µg/mL (Table 2). 

One of the most interesting observations is that the hydrogenated double bond in the prenyl group increases antibacterial activity, as can be observed on the example of inactive isoxanthohumol (**11**) and active 2″,3″-dihydroisoxanthohumol (**14**). A similar effect occurs with hydrogenated α,β-double bond in the chalcone skeleton: α,β-dihydroxanthohumol (**7**) is more active than xanthohumol (**1**). In turn the combination of thereof, thus hydrogenated double bond in the prenyl group and in the α,β-position, in 2″,3″,α,β-tetrahydroxanthohumol (**8**)—increases activity against *S. aureus* ATCC19095, and decreases activity against the tested strains of *S. epidermidis* (Table 2).

Considering the MIC80 values, particularly for methicillin-resistant *Staphylococcus* strains the most promising antimicrobial seems to be natural α,β-dihydroxanthohumol (**7**). Although it is present in hops in amounts less than xanthohumol (**1**), the methods of the selective reduction of **1** to **7** have already been developed [32]. It is worth mentioning that **7** also exhibits slightly higher antitumor activity than **1** and chemotherapeutic drug cisplatin against the breast cancer cell line (MCF-7) [33].

### 3.2. Antifungal Activity

Recently, there is a growing interest in the research of the possible use of natural products, such as plant extracts, which may be less damaging for fungal pathogens control. The antifungal activity of plant extracts is well documented and it is postulated to be the effect of their major compounds or a synergistic effect of various compounds forming a mixture [34].

In the present study, seven field and/or storage phytopathogenic fungal strains from four different species were used to evaluate the possible activity of the spent hops extracts obtained. Apart from the production losses, the *Fusarium* and *Aspergillus* species used produce mycotoxins that pose a health hazard to humans and animals.

The type I extracts limited the growth of fungi with inhibition rates equal to or greater than 50% in seven experiments. Fungi of the genus *Fusarium* were more sensitive to the extracts obtained using the three more polar solvents: ethyl acetate, acetone and methanol, while *Botrytis cinerea* AM235 was significantly inhibited by the crude extract obtained using methylene chloride, the most non-polar extractant used (Table 3).

The most sensitive fungus was *Fusarium culmorum* AM10, as the growth inhibition for the crude extracts obtained with ethyl acetate, acetone and methanol was over 50% (53.1–55.9%). The corresponding type II extracts also showed a high inhibitory effect on *Fusarium culmorum* AM10, their growth inhibition ranged between 41.4–45.2%. It seems that to control these fungal species the residues obtained after the recovery of xanthohumol (**1**) from the raw spent hops extracts can be used. This valuable compound of potential medical use was most effectively isolated with acetone and ethyl acetate (Table 1). It is noteworthy that the antifungal activity of the methanol extracts is almost equal to those obtained using ethyl acetate or acetone, wherein methanol extraction yield is up to 3 times greater.

The obtained extracts weakly inhibited the growth of *Penicillium purpurogenum* AM80 and *Mucor hiemalis* AM450. The maximal growth inhibition observed was 39.3% for *M. hiemalis* and methylene chloride extract I. The removal of xanthohumol (**1**) from the crude extracts in most trials resulted in a total lack of activity (Table 3).

By analyzing the MIC50 values determined for the four most sensitive fungi (Table 4), it can be seen that there was no direct relationship between the content of **1** in extracts and antifungal activity towards the examined fungi. *Fusarium culmorum* AM10 was most susceptible to crude ethyl acetate, acetone and methanol spent hops extracts (MIC50 0.5 mg/mL) and also to pure xanthohumol (**1**) (MIC50 0.015 mg/mL). Although xanthohumol (**1**) content is higher in the methylene chloride extract, the methanol extract was more effective, which may be due to the synergistic effects of **1** with the more polar compounds extracted by this solvent. Ethyl acetate type I extract was the most effective in the suppression of the mycelial growth of *Fusarium oxysporum* AM21 (MIC50 1 mg/mL), which was the most xanthohumol-resistant of the tested *Fusarium* species (MIC50 > 1mg/mL). 

The most interesting area of application for the plant extracts is the inhibition of growth of the more serious plant pathogens like *Botrytis cinerea* and *Fusarium oxysporum*, which are on the ‘Top 10’ fungal plant pathogen list based on scientific/economic importance, published by Molecular Plant Pathology [35]. 

## 4. Materials and Methods

### 4.1. Materials

Extractants: methylene chloride, ethyl acetate, acetone and methanol of analytical grade were purchased from POCh (Gliwice, Poland). Acetonitrile of analytical grade were purchased from Merck (Darmstadt, Germany). Sephadex LH20, formic acid of analytical grade amphotericin B, ampicillin, Müller-Hinton agar and broth were purchased from Sigma—Aldrich (Chemie GmbH, Steinheim, Germany). The spent hops were obtained from the Production of Hop Extracts of New Chemical Syntheses Institute, Puławy, Poland by supercritical carbon dioxide extraction according to Jackowski et al. [36]. Cones (hops) of *Humulus lupulus* cv. ‘Magnum’ collected in 2015 in Lublin region (SE Poland) were used.

### 4.2. Preparation of the Extracts

Spent hops extracts type I: Four solvents with different polarities were used, i.e. methylene chloride, ethyl acetate, acetone and methanol. Spent hops (250 g) were shaken with 1 L of each solvent for 24 h on a rotary shaker. Extracts were filtered with Whatman filter paper 1. Plant material was washed with a portion of 400 mL of solvent and collected filtrates were evaporated under vacuum at the temperature below 50 °C. The residues obtained were stored at −20 °C for further use.

Spent hops extracts type II: spent hops extracts type I were chromatographed on a Sephadex LH–20 column using methanol as eluent. Fractions contained a majority of xanthohumol (**1**) were removed and the rest fractions were collected, evaporated under vacuum at the temperature below 50 °C. The residues obtained were stored at −20 °C for further use.

The all spent hops extracts were analysed for xanthohumol (**1**) content by HPLC on a Dionex Ultimate 3000 instrument (Thermo Fisher Scientific, Waltham, MA, United States) with a diode array detector (detection at 360 nm wavelength) using the analytical HPLC column Agilent ZORBAX Eclipse XDB 5 µm (4.6 × 250 mm). Elution was carried out with a gradient of 40–100% solvent (1% formic acid in MeCN) in solvent A (aqueous 1% formic acid) in 15 min at the flow rate of 0.8 mL/min after initial 2 min at 40% solvent B.

### 4.3. Flavonoids

Flavonoids used in these studies were previously isolated from spent hops, or synthesized. Methods of obtaining these compounds and their spectroscopic data are described in earlier publications [32,37,38,39,40,41,42,43]. Xanthohumol (**1**) was isolated from spent hops according to the procedure described by Tronina et al. [37]. Xanthohumol 4′-*O*-β-d-glucopyranoside (**2**) and xanthohumol 4′-*O*-β-d-(4‴-*O*-methyl)-glucopyranoside (**3**) were obtained by the biotransformation of xanthohumol (**1**) by fungi *Absidia coerulea* and *Beauveria bassiana*, respectively, according to Tronina et al. [38]. 1″,2″-Dihydroxanthohumol C (**4**) was the product of prenyl group cyclisation in xanthohumol (**1**) molecule. Reaction was catalyzed by aluminum chloride (III). Xanthohumol C (**5**) was obtained by cyclisation of xanthohumol (**1**) using 2,3-dichloro-5,6-dicyan-1,4-benzochinone (DDQ). 1″,2″-Dihydroxanthohumol K (**6**) was obtained by cyclisation of xanthohumol (**1**) using trifluoroacetic acid [39]. α,β-Dihydroxanthohumol (**7**) was prepared from xanthohumol (**1**) according to the procedure of regioselective hydrogenation described by Popłoński et al. [32]. α,β,2′,3′-Tetrahydroxanthohumol (**8**) was prepared analogously to α,β-dihydroxanthohumol (**7**), except that the catalyst used was 10% palladium on carbon. 2″-(2‴-Hydroxyisopropyl)-dihydrofurano-[2″,3″:3′,4′]-4′,2-dihydroxy-6′-methoxy-α,β-dihydrochalcone (**9**) was obtained by the biotransformation of α,β-dihydroxanthohumol (**7**) by fungi *Aspergillus ochraceus* according to the procedure described for transformation of **1** by Tronina et al. [40]. α,β,1″,2″-Tetrahydroxanthohumol K (**10**) was prepared analogously to α,β-dihydroxanthohumol (**7**), except that the substrate used was 1″,2″-dihydroxanthohumol K (**6**) [39]. Isoxanthohumol (**11**) was obtained by cyclisation of xanthohumol (**1**) in an alkaline environment by the method described by Bartmańska et al. [41]. Isoxanthohumol 7-*O*-β-d-glucopyranoside (**12**) and isoxanthohumol 7-*O*-β-d-(4‴-*O*-methyl)-glucopyranoside (**13**) were obtained by the biotransformation of isoxanthohumol (**11**) by fungi *Absidia coerulea* and *Beauveria bassiana*, respectively, according to Bartmańska et al. [41]. 2″,3″-Dihydroisoxanthohumol (**14**) was prepared analogously to α,β-dihydroxanthohumol (**7**), except that the substrate used was isoxanthohumol (**11**). 2″,3″-Dihydroisoxanthohumol 7-*O*-β-d-glucopyranoside (**15**) and 2″,3″-dihydroisoxanthohumol 7-*O*-β-d-(4‴-*O*-methyl)-glucopyranoside (**16**) were obtained by the biotransformation of 2″,3″-dihydroisoxanthohumol (**14**) by fungi *Absidia coerulea* and *Beauveria bassiana*. 8-Prenylnaringenin (**17**) was obtained according to the procedure of demethylation of isoxanthohumol (**11**) described by Anioł et al. [42]. 8-Prenylnaringenin 7-*O*-β-d-glucopyranoside (**18**) and 8-prenylnaringenin 7-*O*-β-d-(4‴-*O*-methyl)-glucopyranoside (**19**) were obtained by the biotransformation of 8-prenylnaringenin (**17**) by fungi *Absidia coerulea* and *Beauveria bassiana*, according to Bartmańska et al. [43]. A mixture of 4′,5-dihydroxy-6″,6″-dimethyl-5″,6″-dihydro-4″H-pyrano[2″,3″:7,8]flavanone (**20a**) and 4′,5-dihydroxy-6″,6″-dimethyl-5″,6″-dihydro-4″H-pyrano[2″,3″:6,7]flavanone (**20b**) was the product of prenyl group cyclisation and further demethylation of isoxanthohumol (**11**) molecule. Reaction was catalyzed by aluminum chloride (III). 4′-Hydroxy-5-methoxy-6″,6″-dimethyl-5″,6″-dihydro-4″*H*-pyrano[2″,3″:7,8]flavanone (**21**) was the product of prenyl group cyclisation in isoxanthohumol (**11**) molecule in a reaction catalyzed by aluminum chloride (III). 5-*O*-β-d-Glucopyranosyl-4′-hydroxy-6″,6″-dimethyl-5″,6″-dihydro-4″*H*-pyrano[2″,3″:7,8]flavanone (**22**) was obtained by the biotransformation of the mixture of compounds **20a** and **20b** by fungi *Absidia coerulea*39. Naringenin (**23**) was purchased from Sigma-Aldrich. 2,3-Dehydroisoxanthohumol (**24**) was a product of isoxanthohumol (**11**) dehydrogenation with iodine-pyridine complex [39]. 4′-Hydroxy-5-methoxy-6″,6″-dimethyl-5″,6″-dihydro-4″*H*-pyrano[2″,3″:7,8]flavone (**25**) was prepared analogously to 1″,2″-dihydroxanthohumol C (**4**), except that the substrate used was 2,3-dehydroisoxanthohumol (**24**). (*Z*)-6,4′-Dihydroxy-4-methoxy-7-prenylaurone (**26**) was prepared according to Tronina et al. [37].

### 4.4. Microorganisms

The reference bacterial strains of *Staphylococcus aureus* ATCC19095 (MSSA, enterotoxigenic), *Staphylococcus aureus* ATCC29213 (MSSA), *Staphylococcus aureus* ATCC43300 (MRSA), *Listeria monocytogenes* ATCC7644 and *Salmonella typhimurium* PCM2565 as well as two isolates: *Staphylococcus epidermidis* 91M (MRSE) and *Staphylococcus epidermidis* 4s (MSSE) from the collection of Department of Food Hygiene and Consumer Health Protection, Wrocław University of Environmental and Life Sciences, Poland were used. The phytopathogenic filamentous fungi used in this work, i.e., *Fusarium culmorum* AM10, *Fusarium equiseti* AM15, *Fusarium semitectum* AM20, *Fusarium oxysporum* AM21, *Penicillium purpurogenum* AM80, *Botrytis cinerea* AM235, *Mucor hiemalis* AM450 and *Aspergillus ochraceus* AM456 were obtained from the collection of the Department of Biology and Pharmaceutical Botany, Medical University of Wrocław, (Wrocław, Poland).

### 4.5. Determination of Antibacterial Activity

The first screening of antibacterial activity was carried out using diffusion tests in Müller-Hinton agar medium, according to EUCAST recommendations (Version 1.0, 18 December 2009). The experiment was carried out on three strains: *Staphylococcus aureus* ATCC19095, *Listeria monocytogenes* ATCC7644 and *Salmonella typhimurium* PCM2565. Spent hops extracts and pure flavonoids were dissolved in dimethyl sulfoxide and examined at 100 and 50 μg/mL concentration dose. The inoculum was adjusted to the density 0.5 of a McFarland standard and 100 µL was used for the plate. Then antimicrobials were aseptically dispensed into wells prepared using sterile pipette tips. The inhibition was determined after incubation at 37 °C for 18 h. The negative control with DMSO (5%) and positive control with ampicillin (2.5, 5 and 10 μg) were included.

MIC80 at the range from 5 µg/mL to 50 µg/mL using microbroth dilution test was determined. The panel of the tested microorganisms was expanded to include four more bacterial strains: *S. aureus* ATCC29213 (MSSA), *S. aureus* ATCC43300 (MRSA), *S. epidermidis* 91M (MRSE) and *S. epidermidis* 4s (MSSE, enterotoxigenic). Single colonies were grown in MH broth overnight at 37 °C. Then bacteria were diluted to OD_600_ = 0.01 and cultivated until final concentration of 6 log CFU. The volume of 100 μL of bacterial suspension was added into the well of 96-well plate containing 100 μL of serially diluted antimicrobials. The negative (plain medium) and positive control (bacteria without antimicrobials) were performed. Plates were covered with lid and incubated at 37 °C for 18 h. The MIC endpoint was determined as the lowest concentration of the antimicrobial at which turbidity was lower or equal to 20% of the control (1:5 of control). Turbidity was read at 620 nm. The assay was performed in triplicate with two biological repeats.

### 4.6. Determination of Antifungal Activity

The proper amounts of tested extracts were dissolved in DMSO (0.007 mL of DMSO for 1 mL of agar medium) and added to sterilized Sabouraud agar in Petri dishes. Discs with fungal mycelia (0.4 cm in diameter) were placed on Petri dishes, which were then incubated at 27 ± 2 °C. All the extracts were tested at a concentration of 1 mg/mL. For the experimental arrangements: The most active extract—the most sensitive fungus, three more concentrations were tested, which were chosen individually. Each test was repeated three times. Amphotericin B was used as reference compound. The percent of mycelia growth inhibition was estimated using as reference the control treatment (Petri dishes with Sabouraud agar medium and 0.007 mL of DMSO for 1 mL of agar) as follows: (C − T) / C × 100, where C is the colony diameter under the control treatment and T is the colony diameter under the extract treatment. 

## 5. Conclusions

Our data confirm the findings of the previous studies, which reported that hop prenylated flavonoids and extracts from hops/spent hops exhibit antistaphylococcal activity. However, this is the first study to provide data about the antimicrobial activity of minor hops flavonoids (other than isoxanthohumol, 6- and 8-prenylnaringenin) and some derivatives of xanthohumol obtained by chemical methods. In general, phytochemicals are much weaker than antibiotics, whereas the MIC80 values of some hop flavonoids determined by us were less than 2.5 times weaker against methicillin-resistant *Staphylococcus aureus*. These results indicate that spent hops minor flavonoids are excellent candidates for further research into their uses for food preservation as well as natural pharmaceutical products.

Taking into account the results of the present study, the ethyl acetate, acetone, and methanol crude spent hops extracts, as well as the extracts formed after the removal of xanthohumol, can be used as natural agents for crop protection against various *Fusaria*, while methylene chloride extract may be used against *Botrytis cinerea*. The high activity of these natural formulations obtained from the waste plant material in a simple extraction process makes them economically justified. 

## Figures and Tables

**Table 1:** The content of xanthohumol (**1**) achieved by HPLC in the investigated spent hops extracts type I (obtained by solvent extraction) and type II (obtained from extracts type I by the removal of **1**).

Extracts Type I	Xanthohumol [µg/mg] ^a^	Extracts Type II	Xanthohumol [ng/mg] ^a^
methylene chloride	51.15 ± 0.14	methylene chloride	˂0.5 ^b^
ethyl acetate	90.95 ± 0.28	ethyl acetate	397 ± 15.18
acetone	99.70 ± 0.31	acetone	278 ± 17.95
methanol	31.65 ± 0.60	methanol	1.5 ± 0.91

^a^ Data are mean ± SD (standard deviation); ^b^ value under the limit of quantification.

**Table 2:** Antibacterial activity of hops flavonoids and their derivatives.

Microorganism	MIC80 [µg/mL]						
	7	8	14	17	20a, b	26	Ampicillin
*S. aureus* ATCC19095 (MSSA)	12.5	5	>50	50	12.5	12.5	2.5
*S. aureus* ATCC43300 (MRSA)	12.5	12.5	>50	50	12.5	12.5	˃5
*S. aureus* ATCC29213 (MSSA)	12.5	12.5	50	25	12.5	12.5	˃5
*S. epidermidis* 4s (MSSE)	12.5	>50	50	50	25	12.5	2.5
*S. epidermidis* 91M (MRSE)	25	>50	>50	50	25	25	˃5
*S. typhimurium* PCM2565	na	na	na	na	na	50	5
*L. monocytogenes* ATCC7644	na	na	na	na	na	na	2.5

na—not active at the tested antimicrobials concentration.

**Table 3:** Antifungal activity of the spent hops extracts (type I) and the residues of the spent hops extracts obtained after xanthohumol removing (type II) determined at 1 mg/mL concentration.

Microorganism	Growth Inhibition [%]							
	Methylene Chloride		Ethyl Ccetate		Acetone		Methanol	
	Extract I	Extract II	Extract I	Extract II	Extract I	Extract II	Extract I	Extract II
*F. culmorum* AM10	37.5 ± 0.79	34.2 ± 0,40	55.9 ± 0.79	41.4 ± 0.39	54.6 ± 0.39	45.2 ± 0.79	53.1 ± 0.40	41.4 ± 1.04
*F. equiseti* AM15	35.3 ± 1.50	20.5 ± 2.04	39.7 ± 1.50	16.5 ± 1.13	44.2 ± 0.57	16.9 ± 1.68	41.2 ± 0.98	32.5 ± 0.98
*F. semitectum* AM20	43.2 ± 0.5	15.2 ± 1.29	51.3 ± 0.98	16.2 ± 0.98	54.1 ± 0.85	24.6 ± 0.98	47.3 ± 2.59	31.6 ± 1.76
*F. oxysporum* AM21	31.6 ± 2.68	6.1 ± 0.45	50.0 ± 0.45	7.8 ± 0.77	39.5 ± 0.45	5.1 ± 0.77	36.8 ± 0.45	4.7 ± 1.18
*B. cinerea* AM235	50.9 ± 1.17	25.4 ± 0.88	30.9 ± 1.17	5.3 ± 0.76	36.4 ± 0.44	12.1 ± 0.44	22.4 ± 1.93	21.4 ± 1.59
*P. purpurogenum* AM80	14.3 ± 1.95	0.0 ± 0.98	26.2 ± 1.95	9.9 ± 0.97	23.8 ± 1.69	9.1 ± 0.97	26.2 ± 0.98	1.0 ± 0.98
*M. hiemalis* AM450	39.3 ± 0.6	0.0 ± 1.00	28.9 ± 1.00	0.0 ± 0.50	30.0 ± 1,72	0.0 ± 0.50	30.4 ± 1,79	0.0 ± 1.00

**Table 4:** The MIC50 of crude extracts against the fungal test strains.

Antifungals	MIC50 [mg/mL]			
	*F. culmorum* AM10	*F. semitectum* AM20	*F. oxysporum* AM21	*B. cinerea* AM235
methylene chloride extract I	˃1	˃1	˃1	1
ethyl acetate extract I	0.5	1	1	˃1
acetone extract I	0.5	1	˃1	˃1
methanol extract I	0.5	˃1	˃1	˃1
xanthohumol	0.015	0.030	0.100	˃0.200
amphotericin B	0.005	0.005	0.005	0.005
